# Supplementary material for: Essential role for SUN5 in anchoring sperm head to the tail
Source: eLife. 2017 Sep 25;6:e28199. doi: 10.7554/eLife.28199 (PMC5634783; doi:10.7554/eLife.28199)
Supplement: Figure 4—source data 1. [file elife-28199-fig4-data1.docx]

Figure 4- source data 1. The sperm motility and morphology analysis of the two patients underwent ICSI.

| Patient | Sperm volume (ml) | Concentration  (10^6^/ml) | Motility  a/b/c/d (%) | Percentage of spermatozoa morphology (%) | | | |
| --- | --- | --- | --- | --- | --- | --- | --- |
|  |  |  |  | Normally formed | Abnormal head-tail junction | Decaudated | Acephalic |
| P1 | 2.2 | 5.3 | 0/4.8/18.6/76.6 | 0 | 3.9 | 0.4 | 95.7 |
| P8 | 2.8 | 4.9 | 0/6.1/20.3/73.6 | 0 | 3.3 | 0.1 | 96.6 |

The sperm motility and the percentages of morphologically normal and abnormal spermatozoa were evaluated according to the World Health Organization (WHO) guidelines. Most of the *SUN5*-mutation associated spermatozoa were acephalic sperms with low motility.
