# Supplementary material for: Essential role for SUN5 in anchoring sperm head to the tail
Source: eLife. 2017 Sep 25;6:e28199. doi: 10.7554/eLife.28199 (PMC5634783; doi:10.7554/eLife.28199)
Supplement: Transparent reporting form [file elife-28199-transrepform.docx]

***eLife’s*transparent reporting form**

We encourage authors to provide detailed information *within their submission* to facilitate the interpretation and replication of experiments. If you have any questions, please contact us:[editorial@elifesciences.org](mailto:editorial@elifesciences.org).

**Sample-size estimation**

- You should state whether an appropriate sample size was computed when the study was being designed
- You should state the statistical method of sample size computation and any required assumptions
- If no explicit power analysis was used, you should describe how you decidedwhat sample (replicate) size (number) to use

Please outline where this information can be found within the submission (e.g., page numbers or figure legends), or explain why this information doesn’t apply to your submission:

No statistical method was employed to predetermine the size of the sample. Statistical analysis was performed using 5 independent animals of each genotype, these information could be found in related figure legends.

**Replicates**

- You should report how often each experiment was performed
- You should includea definition of biological versus technical replication
- The data obtained should be provided and sufficient information should be provided to indicate the number of independent biological and/or technical replicates
- If you encountered any outliers, you should describe how these were handled
- Criteria for exclusion/inclusion of data should be clearly stated
- High-throughput sequence data should be uploaded before submission, with a private link for reviewers provided (these are available from both GEO and ArrayExpress)

Please outline where this information can be found within the submission (e.g., page numbers or figure legends), or explain why this information doesn’t apply to your submission:

For the phenotype in this study is not age-related, so all the experiments were performed using adult mice (8-12 weeks old), except for developmental analysis of mice of different genotypes. In Figure supplement 2, mouse body weight were measured in 2 weeks, 4 weeks, 8 weeks, 12 weeks, 16 weeks after birth, respectively. In Figure supplement 6, mouse body weight were measured in 1 weeks, 2 weeks, 4 weeks, 6 weeks, 8 weeks after birth, respectively.

Each independent animal of the same genotype in the analysis was identified as a biological replication, and repeated measurements (5 times usually, especially sperm count, sperm motility and spermatozoa component in Figure 1) of the same animal was identified as technical replication. The number of the replications has been provided in the related figure legends.

**Statistical reporting**

- Statistical analysis methods should be described and justified
- Raw data should be presented in figures whenever informative to do so (typically when N per group is less than 10)
- For each experiment,you should identify the statistical tests used, exact values of N, definitions of center, methods of multiple test correction, and dispersion and precision measures (e.g., mean, median, SD, SEM, confidence intervals; and, for the major substantive results, a measure of effect size (e.g., Pearson's r, Cohen's d)
- Report exact p-values wherever possible alongside the summary statistics and 95% confidence intervals. These should be reported for all key questions and not only when the p-value is less than 0.05.

The statistical significance of the differences between the mean values for the different genotypes was measured by Student’s t-test with a paired, 2-tailed distribution. The data were considered significant when the P value was less than 0.05 (*) or 0.01 (**). The number of N, important values of measurements were provided in figure legends of the related figures.

Please outline where this information can be found within the submission (e.g., page numbers or figure legends), or explain why this information doesn’t apply to your submission:

(For large datasets, or papers with a very large number of statistical tests, you may upload a single table file with tests, Ns, etc., with reference to page numbers in the manuscript.)

**Additional data files (“source data”)**

- We encourage you to upload relevant additional data files, such asnumerical data that are represented as a graph in a figure, or as a summary table
- Where provided, these should be in the most useful format, and they can be uploaded as “Source data” files linked to a main figureor table
- Include model definition files including the full list of parameters used
- Include code used for data analysis (e.g., R, MatLab)
- Avoid statingthat data files are “available upon request”

Please indicate the figures or tables for which source data files have been provided:

The source data of the MS analysis in this study was provided in supplementary file 1.
